# Genome Sequencing and Analysis of *Yersina pestis* KIM D27, an Avirulent Strain Exempt from Select Agent Regulation

**DOI:** 10.1371/journal.pone.0019054

**Published:** 2011-04-29

**Authors:** Liliana Losada, John J. Varga, Jessica Hostetler, Diana Radune, Maria Kim, Scott Durkin, Olaf Schneewind, William C. Nierman

**Affiliations:** 1 J. Craig Venter Institute, Rockville, Maryland, United States of America; 2 University of Chicago, Chicago, Illinois, United States of America; 3 The George Washington University, Washington, D.C., United States of America; Duke University Medical Center, United States of America

## Abstract

*Yersinia pestis* is the causative agent of the plague. *Y. pestis* KIM 10+ strain was passaged and selected for loss of the 102 kb *pgm* locus, resulting in an attenuated strain, KIM D27. In this study, whole genome sequencing was performed on KIM D27 in order to identify any additional differences. Initial assemblies of 454 data were highly fragmented, and various bioinformatic tools detected between 15 and 465 SNPs and INDELs when comparing both strains, the vast majority associated with A or T homopolymer sequences. Consequently, Illumina sequencing was performed to improve the quality of the assembly. Hybrid sequence assemblies were performed and a total of 56 validated SNP/INDELs and 5 repeat differences were identified in the D27 strain relative to published KIM 10+ sequence. However, further analysis showed that 55 of these SNP/INDELs and 3 repeats were errors in the KIM 10+ reference sequence. We conclude that both 454 and Illumina sequencing were required to obtain the most accurate and rapid sequence results for *Y. pestis* KIMD27. SNP and INDELS calls were most accurate when both Newbler and CLC Genomics Workbench were employed. For purposes of obtaining high quality genome sequence differences between strains, any identified differences should be verified in both the new and reference genomes.

## Introduction

Whole genome sequencing (WGS) has broadened the field of microbial forensics by allowing the identification of bacteria not only to the subspecies level, but also to specific sequence types [1]. With the advent of next generation sequencing (NGS) technologies, the application of whole genome sequencing data promises to be even more applicable since it is much more affordable and rapid than traditional Sanger sequencing. The data generated by NGS can be used to fully understand the lineage of naturally or maliciously constructed disease isolates, and could even yield information about past propagation and culturing conditions due to mutations that occur during passage [2], [3]. For forensic purposes, it is necessary to use sequencing methods that yield very accurate results, as well as bioinformatics tools that reveal the similarities and differences with a reference genome such as single nucleotide polymorphisms (SNPs), insertion and deletion events (INDELs), and differences in tandem repeats (VNTRs). To date, genome resequencing efforts with NGS have been used to study the evolutionary lineage of different strains within a single species [2], [4], [5], or to study the types of adaptations that occur during growth in specific conditions [3]. Furthermore, *Y. pestis* has also been subject of NGS sequencing studies to characterize the phylogeographic diversity within the species [6]. The study found that *Y. pestis* strains can be grouped into 4 specific lineages (1 root and 3 branches), that can be characterized further by lineage-specific SNPs [6]. Although the conclusions from these studies depend on the accuracy of the data, any errors inherent in the technology used can be tolerated in some applications. In contrast, for forensic applications, NGS data must be practically error-free since the data could potentially be used to identify and prosecute perpetrators of criminal or terrorist acts.

*Y. pestis* KIM D27, the attenuated laboratory strain, is an isogenic derivative of KIM 10+ (biovar Mediaevalis) that was passaged until the pigmentation phenotype was lost due to loss of the *pgm* locus [7]. The virulent *Y. pestis* KIM 10+ was originally isolated from a human pneumonic plague infection in Iran in 1961 [8]. The 102 kb *pgm* locus is composed of a pathogenicity island and genes involved in pigment synthesis, and its loss results in the attenuated phenotype [7]. The pathogenicity island contains genes for biosynthesis of yersiniabactin [9] and hemin storage (*hms*) genes [10], that are used for iron scavenging [11], biofilm formation [12], and prevention of damage due to reactive oxygen species [13]. Since loss of the *pgm* locus renders a strain avirulent [14], [15], it is exempt from the US Department of Health and Human Services (HHS) Select Agent rules [16], and is commonly used in the lab as a model for *Yersinia pestis* studies. Prior to this report, the genome sequence of *Y. pestis* KIM D27 has not been reported nor has any comprehensive analysis of the details of its genome alterations relative to the fully virulent KIM 10+ parent been considered as they pertain to avirulent phenotype of KIM D27. This issue has assumed some relevance due to the death of a researcher possibly due to an accidental infection by such an attenuated strain of *Y. pestis* [17].

We took advantage of the fact that the genome sequence of *Y. pestis* KIM 10+ was available since its publication in 2002 [18], to test our NGS platforms for forensic purposes by sequencing KIM D27 genomic DNA. Whole genome sequence of KIM D27 confirms the loss of the *pgm* locus and allowed a detailed comparison with the published KIM 10+ sequence. Initial comparisons indicated that KIM D27 contained numerous SNPs and INDELs, but upon further sequencing and examination, these were shown to be errors in the reference KIM 10+ sequence. However, KIM D27 did possess 2 authentic SNPs and 1 repeat expansion relative to the KIM 10+ parent. The data were used to correct the genome sequence of KIM 10+. It was also concluded that the most accurate sequence for genome comparison purposes results from hybrid 454-Illumina sequencing coupled with the use of multiple bioinformatics tools for identifying differences between genome sequences from closely related strains.

## Materials and Methods

### Genome sequencing and assembly

*Y. pestis* KIM D27 genomic DNA was obtained from Olaf Schneewind at the University of Chicago. A 454 Titanium 8 kb paired-end library was constructed and used for sequencing on a full plate of 454 XLR Titanium platform following manufacturer's instructions (Roche, Branford, CT). The overall paired end ratio was 53.9% and the average coverage of the chromosome was 29x. An Illumina paired-end library was constructed and 100 bp sequencing was conducted on Illumina Genome Analyzer IIx following manufacturer's instructions (Illumina, San Diego, CA). Raw sequences from 454 only, 454 and Illumina, or 454, Illumina and Sanger from PCR verification products, were assembled *de novo* using the 454 Newbler assembler [19] and the Celera Assembler 6.1 [20], with default parameters. In addition, mapping assemblies using the published KIM 10+ (accession no. NC_004088) were performed with 454 Newbler, CLC Genomics Workbench (CLC bio, Cambridge, MA), or Amoscmp -shortReads [21] using default parameters. Mate pair information was used to predict the order and orientation of the contigs, confirming that no genomic rearrangements were present relative to KIM 10+.

### Annotation

The KIM D27 genome was annotated through JCVI's auto-annotation pipeline. Whenever possible, annotation followed the nomenclature of KIM 10+. All ORFs ≥ 30 amino acids in length and annotated in other *Yersinia* genomes were added to the KIM D27 annotation.

### SNP, INDELS, and VNTR analysis

The resulting hybrid 454, Illumina, and Sanger assembly was used to align against the *Y. pestis* KIM 10+ nucleotide sequence in CLC Genomics Workbench or Newbler assembler to detect single base-pair polymorphisms (SNP) or insertion/deletion (INDELS) using a 4 read minimum and 2000 read maximum coverage with a frequency of at least 35%, allowing for at most 3 different variants at any site. The results were filtered to exclude any SNPs or INDELs that had low confidence after assembly. All remaining changes were verified by PCR amplification and Sanger sequencing using primers listed in Table S1. SNP or INDELS were corrected to match Sanger read if it did not match the consensus sequence. Repeats were detected with REputer [22] using default parameters and variations in tandem repeat numbers were characterized with Tandem Repeat Finder [23] and CLC genomics workbench. Errors in the *Y. pestis* KIM 10+ genome sequence were identified by PCR amplification and Sanger sequencing. KIM 10+ genomic DNA was purchased from Biodefense and Emerging Infections Research Resources Repository (Cat No. NR-2645; Manassas, VA).

### Nucleotide sequence accession numbers

The *Y. pestis* KIM D27 genome was deposited in GenBank under accession number ADDC00000000.

## Results

### Summary of sequencing and assembly results for *Y. pestis* KIM D27

**Figure 1:**
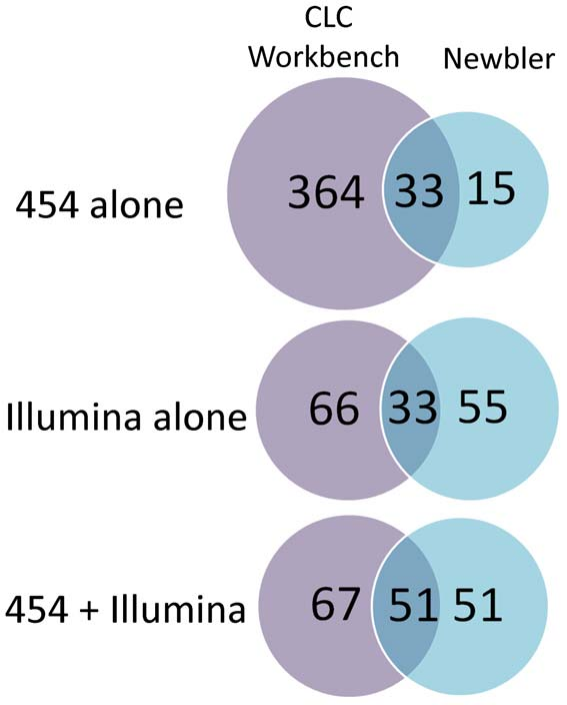
Comparison of SNP and INDELs detection tools based on different sequence assemblies. Assemblies of KIM D27 sequences from either 454 only, Illumina only, or hybrid assemblies (454 + Illumina) were analyzed for SNPs and INDELs as described in Materials and Methods. The Venn diagram represents the number of identified differences in either CLC Genomics Workbench or Newbler either singly (purple or blue, respectively) or the overlap between both programs (darkened area).

The genomic DNA of *Y. pestis* KIM D27 was purified and sequenced on a full-plate of 454 Life Sciences Genome Sequencer FLX machine (Roche, Branford, CT). The 454 sequencing from 8 kb paired end libraries yielded 677,705 reads that passed filter wells with a 53.9% paired end ratio and a total chromosomal coverage of 29X. 454 sequencing data resulted in highly fragmented assemblies using either Celera Assembler 6.1 [20] or Newbler assembler [19], with slightly better results for AMOScmp (12 contigs) [21] (Table 1). The fragmentation was due in part to the complex repeat structure of *Yersinia pestis* genome [24]. The effect of repeats on assembly is evident because 123,000 bases of the KIM 10+ were not covered by a uniquely matching read during reference assemblies, most of these associated with repeat regions or multi-copy sequences like rRNA or transposable elements (not including the known *pgm* deletion discussed below [9]; data not shown). The assemblies showed that the KIM 10+ and KIM D27 genomes had the same overall structure. However, a close inspection of the assembly of 454 sequences resulted in a high number of discrepancies from the KIM 10+ reference genome, including SNPs and INDELs (Figure 1). A majority of these (82%) involved either an A or T residue and most (72%) were primarily associated with poly(A) or poly(T) sequences.

**Table 1:** Summary of sequencing results for Y. pestis KIM D27 and comparison with KIM10.

Sequencing technology	Type of assembly	Total number of contigs	
		*de novo*	Reference
**454**	CA	135	-
	Newbler	306	126
	AMOScmp	−	12
	CLC	−	−
**Illumina**	Newbler	−	−
	CLC	378	4
**454 + Illumina Hybrid**	Newbler	−	120
	AMOScmp	−	1
	CLC Reference	−	4

Because of the large number of SNPs and INDELs identified, additional sequencing was performed with a second platform to eliminate technology-specific errors, such as those associated with homopolymer tracts with the 454 platform. One lane of 100 bp Illumina sequencing was done on a paired end library, yielding over 18 million reads and 371X chromosomal coverage. Hybrid mapping assemblies were generated using Newbler reference assembler (120 contigs) [19], or CLC Genomics Workbench (3 contigs; CLC bio, Cambridge, MA) reference assembler and are summarized in Table 1. In our experience, the consensus built by AMOScmp was faulty due to its inability to efficiently handle homopolymeric bases. This assembly, while structurally matching the KIM 10+ reference, contained hundreds of consensus discrepancies with KIM 10+, and thus was not further analyzed.

The sequence of KIM D27 showed that the chromosomal region from 2580534 to 2679386 of KIM 10+ that contains the *pgm* locus and genes involved in iron acquisition was entirely missing as reported previously [9]. The genetic loss was a consequence of genetic rearrangement across insertion (IS) elements IS100 that border the region. Other organisms, such as *Burkholderia mallei,* have also lost virulence due to rearrangements across IS element while cultured in the laboratory [25]–[26]. From the coverage data, the ratio between the plasmids was determined to be 1∶2∶4 for pMT-1, pCD1, and pPCP1, compared to the chromosome, respectively.

### SNP, INDELs, and repeat analysis and confirmation

A major goal for forensic sequencing and other applications requiring high sequence accuracy is to identify both large and small changes in the genome that could explain the source of a strain or have implications for gene function, for example. Therefore, a detailed analysis of SNPs, INDELs, changes in numbers of repeats and gene-scale gains or losses was conducted. SNPs and INDELs were identified for the chromosome and all three plasmids using CLC Genomics Workbench or Newbler and are summarized in Figure 1. Analysis of 454 data alone produced the most SNP and INDELs (364 by CLC alone, 15 by Newbler alone, 33 by both). The Illumina data alone gave fewer SNPs (59 for CLC alone, 55 by Newbler alone, 48 by both). Hybrid assemblies including both types of data resulted in slightly higher SNPs than Illumina data alone (67 by CLC, 51 by Newbler, 51 by both). For all assemblies, alignment to the KIM 10+ reference sequence identified 3–5 additional SNPs and INDELs, leading to a total of 18 unambiguous SNPs and 38 INDELs. All of these SNPs and INDELs were amplified by PCR and confirmed by Sanger sequencing from KIM D27 source DNA.

**Figure 2:**
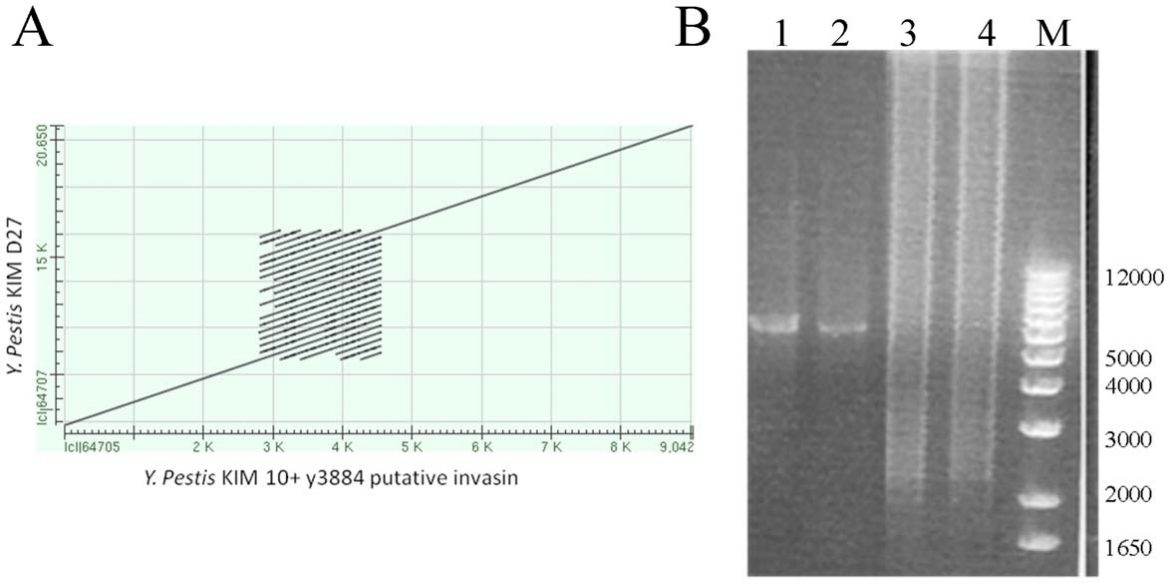
A. Nucmer alignement of putative invasin gene from KIM 10+ and KIM D27. Regions of homology are depicted as diagonal lines. The 289 bp repeat aligns with itself numerous times and results in the square pattern. B. **PCR amplification of the invasin gene from KIM 10+ and KIM D27.** The chromosomal region 4228610 – 4234900 was amplified from genomic DNA from either strain. The predicted 6,290 bp product was observed as the major product from KIM D27 (lanes 1 and 2), but a ladder effect was observed when KIM 10+ DNA was used (lanes 3 and 4).

In addition to SNP/INDELs, Nucmer alignment [27] of KIM D27 *de novo* contigs from the CA6.1 assembly and KIM 10+ identified multiple breaks in regions of suspected differences in numbers of repeat units. REputer [22] analysis showed that 21 of these regions contained repeat elements (e.g., VNTR). After PCR and Sanger sequencing, 16 of these sequences matched the published KIM 10+ sequence. However, 4 of the repeats in KIM D27 appeared to have expanded while 1 repeat had contracted relative to the KIM 10+ published genome (Table 2). Since KIM D27 is a laboratory derived isolate of KIM 10+, the 56 SNP/INDELs and 5 repeat differences seemed suspiciously high, and it was possible that the reference KIM 10+ sequence had some errors. Therefore, *Y. pestis* KIM 10+ DNA was purchased from the Biodefense and Emerging Infections Research Resources Repository (Cat No. NR-2645; Manassas, VA) and used as template for PCR and Sanger sequencing. 55 of 56 SNP/INDELs and 2 repeat expansions and 1 contraction were found to be errors in the KIM 10+ sequence, since the PCR products had the same sequence as KIM D27. Because the trace reads from the KIM 10+ sequence were no longer available we are unable to determine the source of the error. One true SNP between KIM D27 and KIM 10+ was confirmed at coordinate 1505634 on KIM 10+, which results in an Alanine to Valine change in *hisS* (Table 2); one true additional 17 bp tandem repeat expansion was found at coordinate 3346611 upstream of the *modE* gene (Table 3). Lastly, one large expansion (∼3 kb) had occurred in KIM D27 within a gene for a putative outer membrane surface protein (invasin homolog, y3884) that is 9 kb in KIM 10+ and 12 kb in KIM D27 (Table 3 and Figure 2). The difference in size was due to multiple copies of a 289 bp repeat as identified by Tandem Repeat Finder [23]. Interestingly, the KIM 10+ PCR reactions appeared mixed (Figure 2), suggesting that either the genomic DNA was derived from a mixed population of repeats resulting in gene lengths from 7–13 kb or that the repeat is unstable, resulting from copy number variation on replication.

**Table 2:** Repeat expansion or reduction in *Y. pestis* KIM D27.

Type	KIM 10+ coordinates	Size in KIM 10+ (bp)	KIM D27 coordinates	Size in KIM D27 (bp)	Coding	Comment
VNTR	125066–125542	476†	125066–125429	363	N	Tandem repeat. Five copies in KIM 10+; four in KIM D27.
VNTR	704203–704423	220†	704091–704433	342	N	Repeat area expanded in D27.
Expansion*	3346556–3346654	98	3245775–3245890	115	N	17 bp insertion (TTTCTATCTATGTTGTTA) extends N-term of *modE*(y3040)
IS Element	4232115–4232834	719†	4131351–4132781	1430	Y	Tandem repeat of IS154. One copy in reference, two copies in PCR.
VNTR*	4328042–4329222	1180	4227881–4233335	5454	Y	Multiple copies of a 289 bp motif; PCR results in ladder effect in y3884

† Based on published sequence. PCR reactions showed these sites were the same length in KIM 10+ DNA as was observed for KIM D27.

* These sequences were different between KIM 10+ and KIM D27.

**Table 3:** SNP/INDEL confirmed in *Y. pestis* KIM10+.

Type of substitution	Coordinate	Ref	Var	Nearest gene	Annotation	Coding	Result	Homopolymer or repetivite region
SNP (pCD1)	65595	A	G	y0087.1N	putative transposase	N		repetitive region
SNP	121683	A	G	y0111	transposase	Y	No amino acid change	
SNP	142073	C	G	y0129	*gltD;* glutamate synthase beta subunit	Y	P to A at residue 223	4 C
INDEL	276834	−	G	y0258	hypothetical protein	Y	Extends N-term of ORF	3 G
INDEL	327388	G	−	y0305	transposase	N	Possibly extends ORF	
INDEL	655265	−	C	y0579	tyrB; tyrosine biosynthesis	N		
INDEL	887688	−	G	y0795	*mrcB*; murein sacculus	N		3 G
INDEL	897819	A	−	y0800	chloride channel protein	Y	Moves stop 2 aa up	4 A
SNP	988057	G	T	y0879	putative sugar transport	N		
SNP	1086724	T	C	y0962	hypothetical protein	Y	No amino acid change	
INDEL	1176674	G	−	y1043	*tes;* acyl-CoA thioesterase II	N		5 G
SNP	1184200	A	G	y1049	*acrB*; multidrug efflux pump	Y	No amino acid change	6 A
INDEL	1375636	−	C	y1224	NrdE truncation	N	Extends N-term of ORF	7 C
INDEL/SNP	1415467	A	−	y1265	*kdpA;* potassium transporter	Y	Changes balance out	repetitive region
INDEL/SNP	1415469	−	G	y1265	*kdpA;* potassium transporter	Y	Changes balance out	repetitive region
SNP*	1505634	C	T	y1354	hisS	Y	A to V at residue 88	
INDEL	1544038	−	A	y1389	Transcriptional regulator	N		3 A
INDEL	1616527	−	G	y1457	hypothetical protein, putative peroxidase	N		
SNP	1758361	G	T	y1590	*fabB*	N	May change promoter	
INDEL	1830493	C	−	y1655	hypothetical protein	N		
INDEL	2006614	−	C	y1821	mgtA; magnesium transporter	N		repetitive region
INDEL	2006622	A	−	y1821	mgtA; magnesium transporter	N		repetitive region
INDEL	2006743	−	T	y1821	mgtA; magnesium transporter	N		repetitive region
INDEL	2006767	−	T	y1821	mgtA; magnesium transporter	N		repetitive region
INDEL	2006775	−	T	y1821	mgtA; magnesium transporter	N		repetitive region
INDEL	2006778	−	T	y1821	mgtA; magnesium transporter	N		repetitive region
INDEL	2006781	−	T	y1821	mgtA; magnesium transporter	N		repetitive region
INDEL	2006785	−	T	y1821	mgtA; magnesium transporter	N		repetitive region
INDEL	2006791	−	T	y1821	mgtA; magnesium transporter	N		repetitive region
INDEL	2006801	−	T	y1821	mgtA; magnesium transporter	N		repetitive region
SNP	2006807	C	A	y1821	mgtA; magnesium transporter	N		repetitive region
INDEL	2018833	A	−	y1834	hypothetical proteins	Y	Unites 2 ORFs	3 A
SNP	2021584	C	T	y1834	hypothetical protein	Y	No amino acid change	
SNP	2078371	C	T	y1880	hypothetical protein	Y	K to N at residue 33	
INDEL	2253705	G	−	y2047	Tryptophan synthase alpha subunit	Y	Shorter C-term of *trpA*	
INDEL	2377088	A	−	y2150	*purU*	N	Extends N-term of ORF	3 A
INDEL	2377158	T	−	y2150	purU	Y	2nd aa, restores reading frame due to other *purU* change	4 T
SNP	2470103	A	C	y2242	chaperone	Y	No amino acid change	
INDEL	2563999	−	A	y2328	hypothetical protein	Y	Another stop available	5 A
INDEL	2564024	−	G	y2328	hypothetical protein	Y	Another stop available	3 G
SNP	2786829	G	A	y2524	ftn	Y	No amino acid change	repetitive region
SNP	2786831	G	A	y2524	ftn	Y	S to F at residue 11	repetitive region
SNP	2786834	G	A	y2524	ftn	Y	A to G at residue 10	repetitive region
INDEL	2959407	G	−	y2681	hypothetical protein	Y	Joins both ORFs	3 G
SNP	2978605	A	G	y2697	hypothetical protein	Y	V to G at residue 289	
SNP	2981487	C	T	y2698	hypothetical protein	Y	No amino acid change	
SNP	2981565	C	G	y2698	hypothetical protein	Y	No amino acid change	
INDEL	3231270	−	G	y2925	hypothetical protein	N		
SNP	3533369	A	G	y3211	hypothetical protein	Y	N to S at residue 188	
INDEL	3546848	−	A	y3221	PTS permease	Y	Extends ORFs at C-term	
INDEL	3782193	−	C	y3410	Non-ribosomal peptide synthase	Y	Changes balance out	3 C
INDEL	3782201	−	G	y3410	Non-ribosomal peptide synthase	Y	Changes balance out	4 G
INDEL	3782212	A	−	y3410	Non-ribosomal peptide synthase	Y	Changes balance out	
INDEL	3824911	C	−	y3437	hypothetical protein	Y	Joins both ORFs	
INDEL	4154719	G	−	y3736	NadR disrupted	Y	Functional NadR	3 G
INDEL	4363489	G	−	y3907	hypothetical protein	Y	Joins both ORFs	5 G
INDEL	4470283	T	−	y4034	phosphoethanolamine transferase	Y	No amino acid change	

Tables 2 and 3 show the effects of the corrections to the KIM 10+ genome annotation as a result of the identified errors. 23 of the 55 sequence corrections occurred in non-coding regions of the chromosome, while 9 others were silent substitutions within coding regions. 7 of the corrections result in amino acid substitutions. Three of the corrections introduce stop codons, although two of these occur within 2 amino acids of the C-terminus of the protein, and thus are predicted to have little effect on the structure or function of the peptide. In every case where the ORF was extended or truncated, the corrected sequence matched the published sequence of other *Yersinia* genomes (data not shown).

### Determination of the source of the errors in the 454-only assembly

In order to ascertain the potential universality of the problems with the 454-only assembly, an analysis of candidate INDELs conflicts from other 454-only genomes sequenced at the JCVI was performed (Table 4). For each species, the closest finished genome available was chosen as the reference. SNPs were not analyzed because the reference genome did not come from the same strain. The genome sequences of query strains were compared with completed reference sequences published in GenBank, and all INDELs that could result from 454 sequencing errors were identified (Table 4). *Y. pestis* had among the highest average coverage (i.e. the number of reads that represent any given nucleotide) and number of INDELs, but all genomes contained potential 454-introduced errors. There was no relation between the number of homopolymeric-INDELs and % GC content of the genome. As expected, in general the higher the sequencing coverage, the lower the number of INDELs (P<0.05), although this was not true for *Y. pestis*.

**Table 4:** Search for conflicting INDELs in 454-only genomes.

Organism	Technology	% Genome Covered	%GC	Average Coverage	Conflicting INDELs	Short Sequence Repeats
***E. coli strain HMP***	paired end	99%	51%	29x	12	57
***P. gingivalis W83***	fragment	100%	48%	18x	21	58
***E. coli strain HMP***	fragment	99%	51%	19x	95	64
***M. tuberculosis strain***	fragment	99%	66%	16x	118	78
***M. tuberculosis strain***	fragment	98%	66%	13x	189	97
***M. tuberculosis strain***	fragment	98%	66%	13x	206	94
***Y. pestis KIM D27***	paired end	98%	48%	29x	411	246

**Figure 3:**
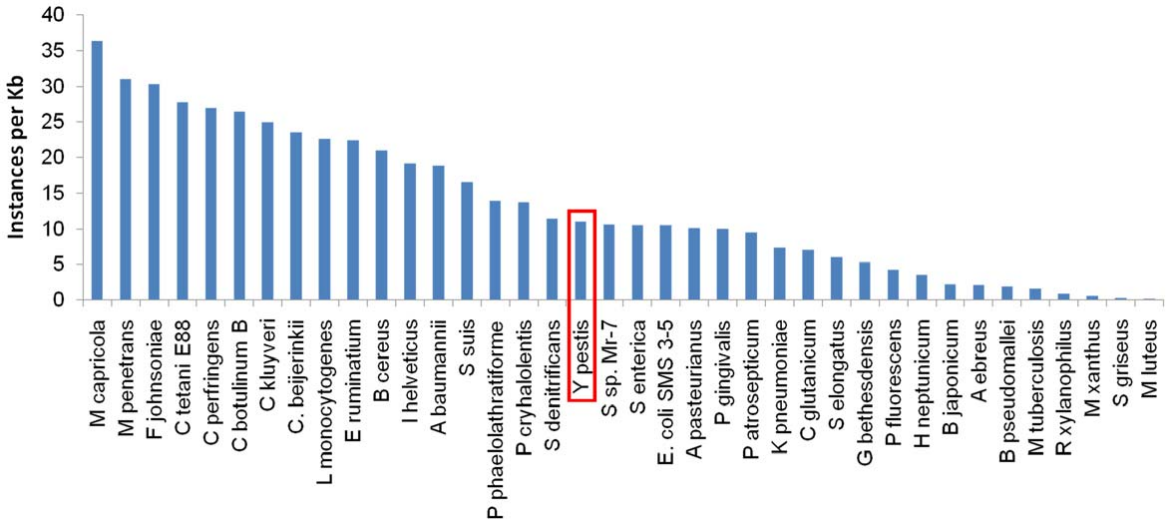
Instances of poly(A) and poly(T) in sequenced genomes. All 4–9 bp poly(A) and poly(T) in published genomes were counted and plotted per kilobase in each genome.

Since the errors were mostly present at poly(A) and poly(T) sequences it is possible that the large number of errors in the KIM D27 454 sequence were due to the strain having an aberrantly high amount of these homopolymer sequences in its genome. The genomes of several species were analyzed for incidence of 4–9 base A or T sequences in CLC Genomics Workbench. *Y. pestis* had an A or T homopolymer frequency of 11.00 per kb, close to the average (13.23) (Figure 3) and an overall number of A or T homopolymers of 49,536 compared to the average of 52,200. This indicates that the number of 454 errors was not simply an effect of having a high frequency or occurrence of A and T homopolymers. However, the number of simple sequence repeats (SSR; i.e., motifs between 2 and 25 nucleotides that are repeated in tandem) had a positive correlation with the number of INDELs (P<0.005), suggesting that genomes with highly repetitive sequences pose significant difficulties for sequencing and assembly (Table 4). However, increasing sequencing coverage and incorporating Illumina data yielded a better genome assembly than either the Sanger only [18], or 454 only data.

## Discussion

In this study we tested our next generation sequencing (NGS) platforms for the purposes of rapidly and accurately sequencing and characterizing disease causing microorganisms. Using the 454 sequencing platform we obtained the genomic sequence of *Y. pestis* KIM D27 within days of obtaining the genomic DNA. After various attempts at assembling the genome, we discovered that 454-only sequencing yielded large numbers of SNP, INDELS, and VNTR differences when compared to the parental genome, most within poly(A) or poly(T) homopolymer tracts. Consequently, we complemented the 454 data with Illumina sequencing data to improve the consensus sequence. We found that optimal sequencing of *Y.pestis* KIM D27 required hybrid assemblies of 454 paired end data and Illumina reads. The hybrid assembly also resulted in highly effective SNP identification, with neither CLC Genomics workbench nor Newbler making false positive calls. The inclusion of multiple SNP calling programs resulted in no false positives called by more than one tool, providing the most confidence in the SNPs that were called. In our experience, the primary strengths of 454 sequencing were that the long reads allow *de novo* assembly and detection of structural changes in a genome, and rapid sequencing and assembly (less than 1 week in the case of Y. pestis KIM D27). However, the consensus quality is not optimal due to artifacts in homopolymer tracts, especially in genomes with high numbers of short repeats. In turn, the coverage depth acquired by Illumina sequencing provides very accurate consensus sequence generation and analysis, including mapping assemblies, SNP and INDEL calling. However, Illumina sequencing requires more time to generate data, and in general, *de novo* assemblies are more fragmented than 454-sequence assemblies. We found that the Celera Assembler was able to handle both 454 and hybrid assemblies better and resulted in more robust, accurate assemblies than any of the other programs used. However, mapping assemblies were best performed by CLC Genomics Workbench. We also found that SNP and INDELs were best identified by using several bioinformatic tools, comparing their results, and curating the list of candidate modifications manually.

Analysis of other 454-only genomes from JCVI indicated that, while the severity of the A and T homopolymer error rate was excessive for *Y. pestis,* the problem is likely to be present in all of the genomes. While the problem can be partially obviated by increased 454 sequencing depth, we found that the error rate was not correlated with the level of coverage, but rather with the number of simple sequence repeats present in the genome. Indeed, a recent study using 454 only sequencing of *B. anthracis* found that the number of potential SNP/INDELs variants among 8 derivatives from either the Ames or Sterne lineages could be as high as 97 differences [28], and was not related to the level of coverage. The authors were able to verify roughly one half of those differences as true differences using PCR and Sanger sequencing. However, there still remain one half of those differences that can be attributed to sequencing errors. Thus, it might be more cost effective to add Illumina sequencing instead of additional 454 sequencing.

The *Y. pestis* KIM D27 genome sequence did not reveal any additional genome alterations that would contribute to its attenuated phenotype beyond those already discussed [9], [11], [14], [15]. This analysis, however, revealed that KIM 10+ and to a lesser extent KIM D27, encode a highly variable region within a putative invasin gene (y3040; Figure 2), which may confer adaptation to different hosts, or immune reactions. An additional variable region was detected in the molybdate transport transciriptional regulator, *modE,* which extended the N-terminus of the peptide by 17 residues. A similar extension is observed in *Y. pestis* Antigua and *Y. pestsis* Angola. Interestingly, the additional residues add a secretion signal as predicted by SignalP [29], suggesting there might be a different response to molybdates in KIM D27 than KIM 10+. Lastly, the modification to the *hisS* gene at residue A88V is not predicted to have a major effect on the protein, because valine is not an uncommon residue at that location among members of the HisS family. However, a modification in HisS could result in differences in the histidine biosynthesis pathway regulation between the two strains that could results through adaptation to laboratory growth.

Importantly, the fact that out of the 56 SNPs and INDELs and 5 repeat size changes initially validated in KIM D27, all but 1 SNPs and 2 repeat expansions (Tables 2 and 3) were found to be sequence errors in the reference genome has significant implications for forensic microbiology and applications involving comparisons with published reference genomes. Specifically, newly observed alterations must be analyzed in both the new isolate as well as the reference genome to discard the possibility of errors in published sequences. Our predicted error levels in the recently 454-only sequenced genomes (see Table 3) combined with those found in older Sanger-only genome sequences would make it virtually impossible to achieve high confidence SNP determinations using solely 454-sequence data, eliminating the possibility of definitive conclusions about the source of disease-causing strains in an investigation such as the Anthrax attacks. Additionally, the limitations of observing large chromosomal rearrangements using only Illumina (or other short read technology), obligates the use of a combined sequencing approach for forensic investigations.

## Supporting Information

Table S1 **List of primers used to validate SNPs and INDELs in Yersinia pestis KIM10 and KIM D27.**
(XLS)
